# Complete Mitochondrial Genome of *Acanthosoma murreeanum* (Hemiptera: Acanthosomatidae): Comparative Analysis and Phylogenetic Implications

**DOI:** 10.3390/genes17050560

**Published:** 2026-05-09

**Authors:** Linmei Ye, Tianlai Huang, Laizheng Jiao, Zhihua Lin, Jie Chen

**Affiliations:** 1 Industrial College of Traditional Chinese Medicine and Health, Lishui University, Lishui 323000, China; ylm655169@sina.com (L.Y.); linzhihua1015@126.com (Z.L.); 2 Qingtian County Forestry Technology Extension Station, Lishui 323000, China; htllai@163.com; 3 College of Life Sciences, University of Chinese Academy of Sciences, Beijing 100049, China; jiaolaizheng23@mails.ucas.ac.cn

**Keywords:** *Acanthosoma murreeanum*, *Acanthosoma*, Acanthosomatidae, mitochondrial control region, mitochondrial genome, phylogenetic analysis

## Abstract

**Background:** Acanthosomatidae (Hemiptera: Pentatomoidea), commonly known as parent bugs, is a comparatively small pentatomoid family whose biological distinctiveness is exemplified by the repeated evolution of maternal egg–nymph guarding in several lineages; however, mitogenomic data for this group remain limited. *Acanthosoma murreeanum* is an important representative of *Acanthosoma*, yet its complete mitochondrial genome and comparative mitogenomic characteristics have not been comprehensively studied. **Methods:** Here, we obtained the complete mitochondrial genome of *A. murreeanum* through sequencing, assembly, and annotation. We then characterized its mitogenomic structure, nucleotide composition, codon usage, RNA structural features, control-region organization, nucleotide diversity, evolutionary rates, and phylogenetic position. In addition, control-region characteristics were compared among available acanthosomatid mitogenomes to evaluate structural variation in the AT-rich region. **Results:** The sequenced mitochondrial genome of *A. murreeanum* is a circular molecule of 15,718 bp, comprising the standard set of 37 mitochondrial genes and a control region of 1104 bp. The genome exhibits a strong A + T bias (74.04%) and retains the typical mitochondrial gene order without gene rearrangement. Most protein-coding genes start with standard ATN codons, except *COX1*, which begins with TTG, whereas *COX2* and *ND5* terminate with incomplete stop codons. Most predicted tRNA genes displayed the conventional cloverleaf configuration, whereas *trnS1* lacked a complete DHU arm and instead formed a simple loop. The control region was characterized by a 60 bp tandem-repeat unit and several conserved sequence motifs. Comparative analysis showed that control-region length, AT content, repeat-unit size, and motif composition varied among sampled Acanthosomatidae, while *A. murreeanum* and *A. haemorrhoidale* shared similar 60 bp tandem-repeat organization. Among the mitochondrial PCGs, *ATP8* exhibited the highest level of variation, whereas *COX1* was the most conserved. The Ka/Ks values of all genes were lower than 1, suggesting that these genes have evolved under purifying selection. Phylogenetic analyses based on maximum-likelihood and Bayesian-inference methods consistently supported a sister relationship between *A. murreeanum* and *A. haemorrhoidale*. **Conclusions:** This study provides a new mitogenomic resource for Acanthosomatidae and represents the first detailed comparative mitogenomic analysis within *Acanthosoma*. The results suggest that *A. murreeanum* retains a conserved mitochondrial genomic architecture, whereas variation in the AT-rich control region provides additional evidence for lineage-specific mitogenomic differentiation. These results provide useful insights into mitogenome evolution and phylogenetic relationships within *Acanthosoma* and closely related acanthosomatid groups.

## 1. Introduction

Acanthosomatidae (Hemiptera: Pentatomoidea), commonly known as parent bugs, is a comparatively small but biologically distinctive family within Pentatomoidea, comprising approximately 57 genera and more than 200 described species [1]. Members of this family are notable for their characteristic maternal care, especially egg–nymph guarding behavior, and both morphological and molecular evidence support the monophyly of the group [2,3,4]. *Acanthosoma* is one of the best-known and most species-rich genera in the family, distributed mainly in Eurasia, and its taxonomic limits have been substantially clarified in recent years [5,6]. Nevertheless, phylogenetic relationships within Acanthosomatidae, particularly among genera and closely related lineages, remain insufficiently resolved. More broadly, recent molecular studies of Pentatomoidea have continued to refine higher-level classification and suggest that some traditionally emphasized morphological characters may be homoplastic, highlighting the need for additional lineage-focused molecular datasets.

In insects, the mitochondrial genome (mitogenome) is typically a compact circular molecule, approximately 15–20 kb in size, and usually includes 13 protein-coding genes (PCGs), 22 transfer RNA genes, two ribosomal RNA genes, and a control region (CR). Its conserved gene content, maternal inheritance, and strong phylogenetic utility make it a valuable molecular marker for comparative genomics, molecular evolution, and phylogenetic reconstruction. In Pentatomoidea, mitogenomic research has progressed from small exploratory datasets to broader comparative frameworks. A superfamily-scale study including 55 species from eight families revealed substantial compositional heterogeneity and contrasting evolutionary rates among lineages, and further showed that denser taxon sampling and appropriate analytical models are essential for recovering stable phylogenetic relationships [7]. Subsequent studies in Pentatomidae expanded genus- and tribe-level sampling and demonstrated that pentatomoid mitogenomes generally retain conserved gene order, but vary markedly in control-region organization, RNA secondary structures, and gene-specific evolutionary rates; notably, cytochrome oxidase genes tend to be more conserved, whereas *ATP8* is often among the fastest-evolving loci [8,9,10,11].

In contrast to the rapid accumulation of mitogenomic data in Pentatomidae and other pentatomoid families, Acanthosomatidae remains poorly represented in current comparative datasets. In the most taxon-rich Pentatomoidea mitogenomic analysis currently available, only five acanthosomatid taxa were included, indicating that family-level sampling is still limited [7]. As a result, it remains unclear whether the genomic features and gene-wise evolutionary patterns documented in other pentatomoid groups are also characteristic of Acanthosomatidae. Here, we obtained the complete mitochondrial genome of *Acanthosoma murreeanum* and carried out its annotation. We analyzed its gene organization, base composition, codon usage, RNA features, and control-region motifs, and further investigated nucleotide diversity and substitution patterns across mitochondrial PCGs. Phylogenetic relationships within Acanthosomatidae were also reconstructed using mitochondrial datasets. This work adds a new mitogenomic resource for a poorly represented pentatomoid family, provides an additional basis for comparison within *Acanthosoma*, and contributes to a clearer understanding of mitogenome evolution and phylogeny in Acanthosomatidae.

## 2. Materials and Methods

### 2.1. Specimen Collection and Taxonomic Identification

A specimen of *A. murreeanum* was collected from Fragrant Hills, Beijing, China (40.01° N, 116.29° E). After collection, the live specimen was thoroughly rinsed under running water to remove surface contaminants, then transferred to a 15 mL centrifuge tube containing absolute ethanol and stored at −80 °C, until DNA extraction. Species identification was conducted based on the morphological descriptions provided in the taxonomic literature on *Acanthosoma* [6]. A photograph of the specimen was taken using a Nikon Z5 camera (Nikon Corporation, Tokyo, Japan). *A. murreeanum* is not listed as threatened on the IUCN Red List, nor is it included among China’s National Key Protected Wild Animals.

### 2.2. DNA Extraction and Sequencing

Genomic DNA was extracted from the thoracic and leg muscles of a single adult specimen with the EasyPure^®^ Genomic DNA Kit, following the manufacturer’s protocol (TransGen, Beijing, China). DNA quality was examined using 1% agarose gel electrophoresis, and DNA concentration was quantified with a Qubit^®^ DNA Assay Kit and Qubit^®^ 2.0 Fluorometer (Life Technologies, Waltham, CA, USA). Paired-end libraries were then constructed and sequenced on an Illumina NovaSeq 6000 platform by Berry Genomics Co., Ltd. (Beijing, China), yielding 150 bp paired-end reads.

### 2.3. Mitogenome Assembly and Annotation

Raw sequencing reads were filtered and quality-controlled using fastp v1.0.1 [12]. The mitogenome was then de novo assembled from the clean reads using GetOrganelle v1.7.7.1 [13], and the circularity of the assembled genome was subsequently confirmed. Initial annotation of the mitogenome was performed in Geneious Prime 2025.0.2, followed by re-annotation using the MITOS2 web server [14] and MitoZ v3.6 [15]. Gene boundaries, including the start and stop positions of each gene, were manually checked and adjusted in Geneious Prime 2025.0.2.

### 2.4. Sequence Analysis

The base composition of the mitogenome was determined in Geneious Prime 2025.0.2. AT-skew and GC-skew were estimated using the equations AT-skew = (A − T)/(A + T) and GC-skew = (G − C)/(G + C), respectively [16]. Amino acid composition and relative synonymous codon usage (RSCU) patterns of the protein-coding genes (PCGs) were examined and plotted with PhyloSuite v2.0 [17]. The boundaries, anticodons, and secondary structures of tRNA genes were predicted by MITOS2. Nonsynonymous substitution rates (Ka), synonymous substitution rates (Ks), and nucleotide diversity (Pi) for the PCGs were estimated in DnaSP v6.12.03 [18]. The circular mitogenome map of *A. murreeanum* was drawn using the online tool OGDRAW v1.3.1 [19].

### 2.5. Phylogenetic Analysis

Phylogenetic analyses were carried out in PhyloSuite v2.0 [17]. Each PCG was first aligned with MAFFT v7.526 [20], followed by refinement using MACSE v2.06 [21]. Regions with poor alignment quality or ambiguous homology were excluded with Gblocks v0.91b [22]. The two rRNA genes were aligned independently using MUSCLE v5 [23], and the resulting alignments were trimmed with trimAl v1.4.1 [24] under default parameters. All processed alignments were then concatenated in PhyloSuite v2.0. Partitioning strategies and best-fit substitution models for the combined dataset, consisting of 13 PCGs and two rRNA genes, were inferred with ModelFinder implemented in IQ-TREE v3.0.1 [25] according to the corrected Akaike information criterion (AICc) for both maximum-likelihood (ML) and Bayesian-inference (BI) analyses. ML tree reconstruction was implemented in IQ-TREE v3.0.1 [26], with node support assessed by 10,000 ultrafast bootstrap replicates. BI analysis was performed using MrBayes v3.2.7a [27], with two independent runs and four Markov chain Monte Carlo (MCMC) chains per run for 5,000,000 generations, sampling every 1000 generations. Convergence was judged to be adequate when the average standard deviation of split frequencies was less than 0.01. The initial 25% of sampled trees were removed as burn-in.

## 3. Results

### 3.1. Mitogenome Organization and Base Composition

**Figure 1:**
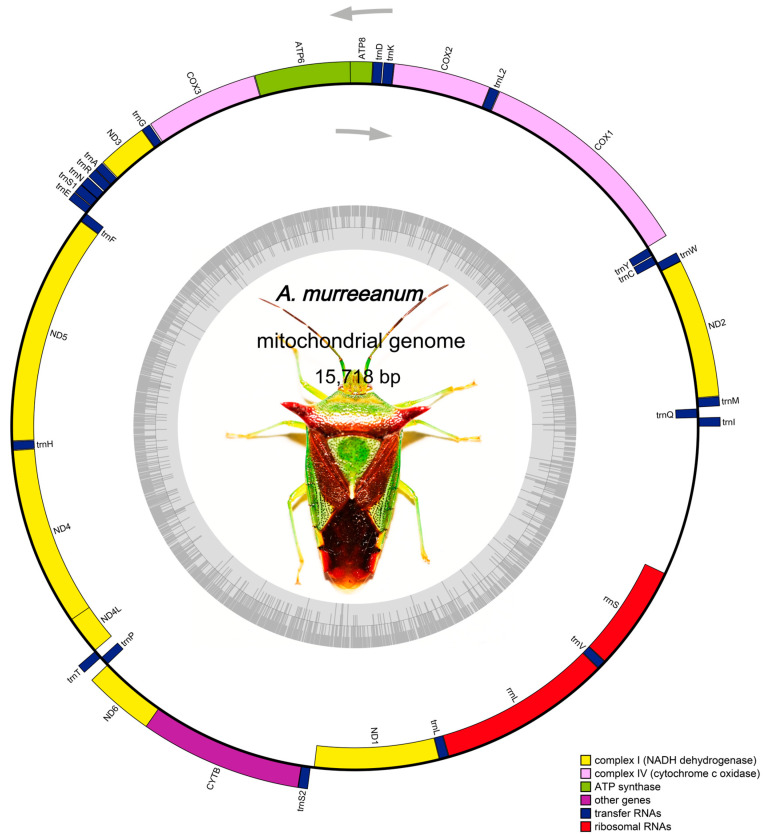
Circular mitogenomic map of *A. murreeanum*. The arrows indicate the direction of gene transcription.

The mitogenome of *A. murreeanum* was assembled as a circular DNA molecule of 15,718 bp in length (Figure 1). A pronounced AT bias was observed across the whole mitogenome, with the total AT content reaching 74.04% (Table 1). Consistent with the typical insect mitogenome, it includes 13 PCGs, 22 tRNA genes, two rRNA genes, and a 1104 bp control region (Figure 1). Among these genes, 23 are encoded on the major strand (J-strand), whereas the remaining 14 occur on the minor strand (N-strand) (Table 2). Among different genomic regions, PCGs (−0.10) and rRNAs (−0.17) showed negative AT-skew, whereas tRNAs (0.03) and the control region (0.04) exhibited positive AT-skew. Similarly, PCGs (−0.02) and the control region (−0.25) displayed negative GC-skew, while tRNAs (0.13) and rRNAs (0.25) showed positive GC-skew (Table 1).

### 3.2. Protein-Coding Genes and Codon Usage

**Figure 2:**
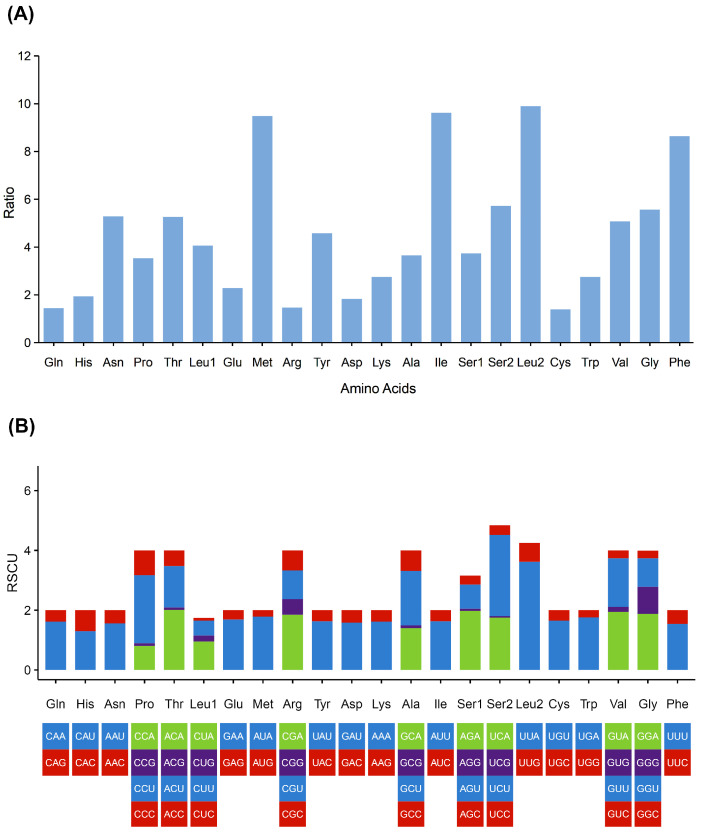
Amino acid profiles and relative synonymous codon usage of mitochondrial protein-coding genes in *A. murreeanum*. (**A**) Amino acid composition, (**B**) relative synonymous codon usage.

The mitogenome of *A. murreeanum* harbors 13 PCGs, which together encode 3668 amino acids. Amino acid usage showed a pronounced bias (Figure 2). The dominant codons were AUA (Met), UUA (Leu2), AUU (Ile), and UUU (Phe), reflecting a marked preference for A and U nucleotides (Figure 2B). The codon profile in the mitogenome of Acanthosomatidae also exhibited a clear bias toward A or U at the third codon position (Figure 2B and Figure S1). The majority of PCGs initiated with standard ATN codons, whereas *COX1* adopted TTG as the start codon. Most genes terminated with the standard stop codons TAA or TAG, while *COX2* and *ND5* ended with an incomplete single-T stop codon (Table 2).

### 3.3. Transfer and Ribosomal RNA Genes

**Figure 3:**
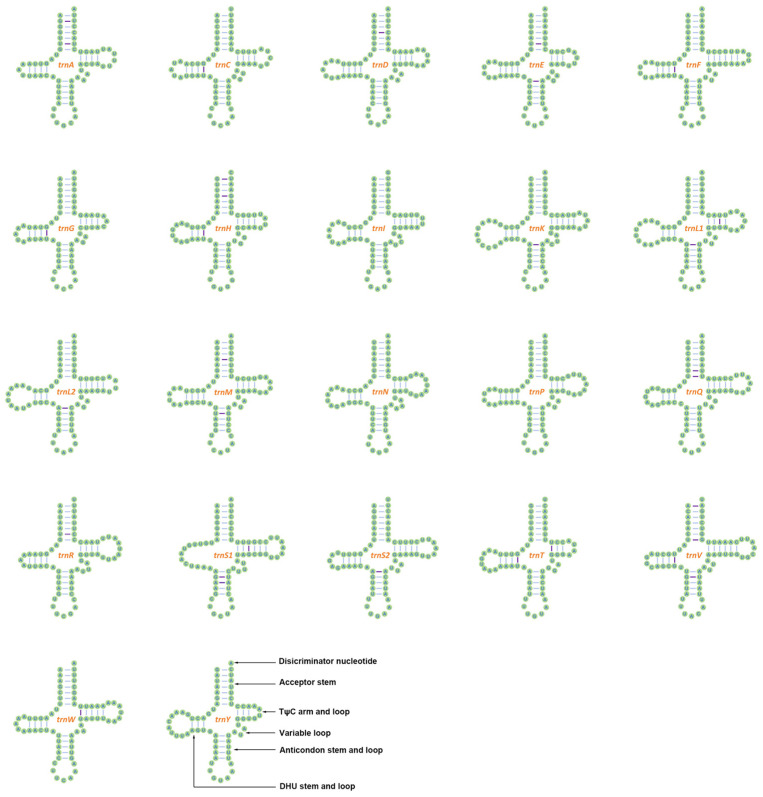
Predicted secondary structures of the 22 mitochondrial tRNA genes in *A. murreeanum*. Purple lines indicate mismatched base pairs.

Twenty-two tRNA genes were found in the mitogenome of *A. murreeanum*, ranging from 63 to 72 bp in length. Among them, 14 occurred on the J-strand, whereas eight were encoded on the N-strand. Predicted structural analysis showed that most tRNAs adopted the conventional cloverleaf structure, except *trnS1*, whose DHU arm was reduced to a simple loop (Figure 3). The two rRNA genes, *rrnL* (1280 bp) and *rrnS* (782 bp), were both positioned on the N-strand and separated by *trnV*. The A + T contents of tRNAs and rRNAs reached 77.46% and 77.88%, respectively.

### 3.4. Comparative Analysis of the Mitochondrial Control Region

The control region of *A. murreeanum* was located between *rrnS* and *trnI*, with a length of 1104 bp and an AT content of 73.55%. Tandem repeats of a 60 bp unit were detected in this region, together with several sequence motifs, including poly(T), poly(A), C(n)GG, (TA)n, and ATAGA. To compare control-region variation within Acanthosomatidae, seven available acanthosomatid mitogenomes were analyzed (Table 3). The control-region length varied markedly among species, ranging from 845 bp in *Microdeuterus* sp. to 4328 bp in *A. haemorrhoidale*. The AT content ranged from 70.89% in *Sastragala esakii* to 79.95% in *A. haemorrhoidale*. Tandem repeats were detected in all sampled species, but their repeat-unit lengths and copy numbers differed among taxa. Notably, *A. murreeanum* and *A. haemorrhoidale*, which formed a sister relationship in the phylogenetic tree, both contained 60 bp tandem-repeat units in the control region.

### 3.5. Nucleotide Diversity and Evolutionary Analysis

**Figure 4:**
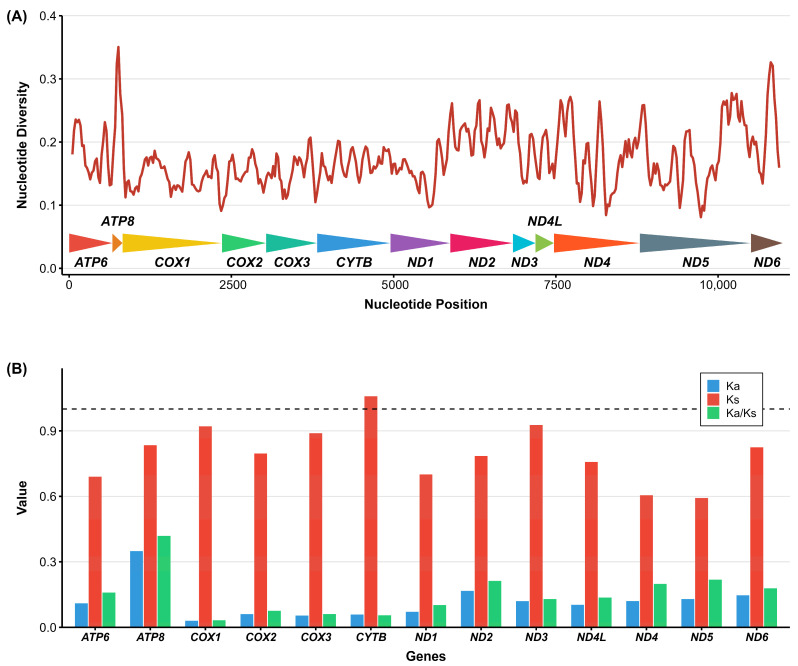
Nucleotide diversity (Pi) and the ratio of nonsynonymous (Ka) to synonymous (Ks) substitution rates of the 13 protein-coding genes within Acanthosomatidae. (**A**) Nucleotide diversity, (**B**) Ka/Ks values. The dashed line in panel B indicates Ka/Ks = 1.

The nucleotide diversity (Pi) of the 13 PCGs varied substantially across the mitogenome of *A. murreeanum* (Figure 4A). Among these genes, *ATP8* showed the highest variability, whereas *COX1* was the most conserved. Pairwise comparisons of Ka, Ks, and Ka/Ks further revealed heterogeneous evolutionary rates among PCGs (Figure 4B). The gene *ATP8* exhibited the highest Ka and Ka/Ks values, while *COX1* showed the lowest values. All Ka/Ks ratios were below 1, indicating that all mitochondrial PCGs are subject to purifying selection.

### 3.6. Phylogenetic Relationships

**Figure 5:**
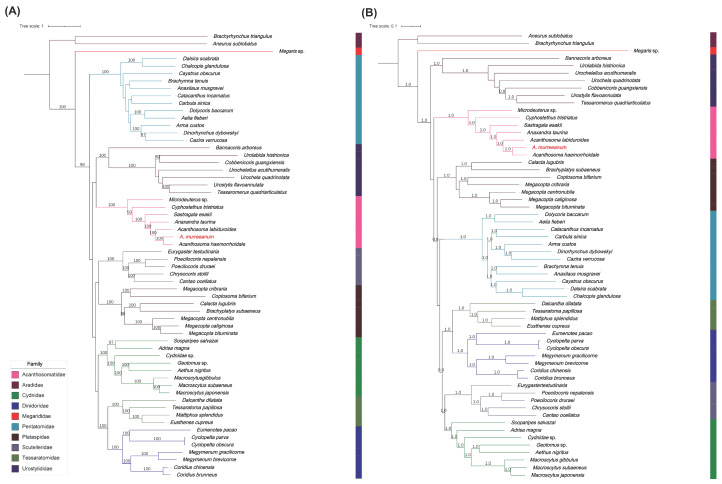
Phylogenetic trees reconstructed from the concatenated matrix of 13 protein-coding genes and two rRNA genes. (**A**) Maximum-likelihood (ML) tree; (**B**) Bayesian-inference (BI) tree. Node values represent ML bootstrap supports and BI posterior probabilities, respectively. *A. murreeanum* is highlighted in red. Details of the sequences included in the phylogenetic analyses are provided in Table S1.

Phylogenetic relationships were reconstructed using maximum-likelihood (ML) and Bayesian-inference (BI) methods based on the concatenated mitochondrial dataset. The ML and BI analyses recovered largely congruent topologies for Acanthosomatidae and *Acanthosoma*. In both analyses, the sampled Acanthosomatidae species formed a strongly supported monophyletic clade. Within Acanthosomatidae, *A. murreeanum* was consistently recovered as the sister species of *A. haemorrhoidale* with strong support (ML bootstrap = 100; BI posterior probability = 1.0). These two species were further grouped with *A. labiduroides*, supporting the monophyly of the sampled *Acanthosoma* species. Other sampled acanthosomatid genera, including *Anaxandra*, *Sastragala*, *Cyphostethus*, and *Microdeuterus*, were placed outside the Acanthosoma clade (Figure 5).

## 4. Discussion

The complete mitogenome of *A. murreeanum* exhibits the general characteristics typically observed in pentatomoid mitogenomes. Its genome size, gene content, strand distribution, and strong A + T bias are all consistent with the conserved mitogenomic pattern reported for Pentatomoidea and Pentatomidae in previous comparative studies [7,8,10,28]. In particular, the overall A + T content of *A. murreeanum* (74.04%) falls well within the range commonly reported for pentatomoid mitogenomes. These results indicate that the mitogenome of *A. murreeanum* is structurally conservative, while also providing an important new dataset for the still underrepresented family Acanthosomatidae. Notably, no gene rearrangement was detected in the mitogenome of *A. murreeanum*, indicating that this species retains the typical mitochondrial genomic architecture of insects. This conserved organization suggests that mitochondrial gene arrangement in *A. murreeanum* may be subject to strong structural constraints, particularly in protein-coding genes, rRNAs, and tRNAs.

The absence of gene rearrangement should not be interpreted simply as a lack of structural novelty. Instead, it provides evidence that mitogenome evolution in *Acanthosoma* may be characterized by stability in coding and RNA gene organization, whereas more dynamic changes occur mainly in non-coding regions. This pattern is consistent with the general evolutionary characteristics of many pentatomoid mitogenomes, in which gene content and gene order are relatively conserved, while variation is more frequently observed in nucleotide composition, codon usage, substitution rates, RNA secondary structures, and the control region [7,8,10,28]. Therefore, the conserved genomic architecture of *A. murreeanum* may reflect evolutionary constraints on mitochondrial genome organization within Acanthosomatidae, while still allowing lineage-specific variation in more flexible regions of the mitogenome.

The coding and RNA features of *A. murreeanum* are likewise comparable to those reported from other pentatomoid insects. Most PCGs initiated with the standard ATN codons, whereas *COX1* used TTG as the start codon, and *COX2* and *ND5* ended with incomplete stop codons. Similar patterns have been widely documented in pentatomoid and other hemipteran mitogenomes [8,28,29]. Codon usage in *A. murreeanum* showed a strong preference for A/U-ending codons, which is clearly associated with the AT-rich nucleotide composition of the mitogenome. Among the 22 tRNAs, only *trnS1* displayed a reduced DHU arm, whereas the remaining tRNAs retained the typical cloverleaf structure. This feature is common in insect mitogenomes and has also been reported in pentatomoid comparative analyses [11,28]. Together with the conserved gene order, these coding and RNA characteristics further support the view that the mitogenome of *A. murreeanum* follows a conservative organizational pattern typical of Pentatomoidea.

In contrast to the conserved gene arrangement, the mitochondrial control region showed marked structural variation among the sampled Acanthosomatidae, consistent with the high variability and tandem-repeat organization reported for hemipteran mitochondrial control regions [30]. In *A. murreeanum*, the control region was 1104 bp in length and contained tandem repeats of a 60 bp unit, together with several simple sequence motifs, including poly(T), poly(A), C(n)GG, (TA)n, and ATAGA. Comparative analysis of seven acanthosomatid mitogenomes showed that the control-region length varied substantially, ranging from 845 bp in *Microdeuterus* sp. to 4328 bp in *A. haemorrhoidale*, and that tandem repeats were detected in all sampled species. However, the repeat-unit length, copy number, and motif composition differed among taxa. Notably, *A. murreeanum* and *A. haemorrhoidale*, which were recovered as sister species in the phylogenetic analyses, both contained 60 bp tandem-repeat units and showed relatively similar repeat organization. Because several short motifs and simple repeats also occurred in other acanthosomatid taxa, these characters should not be regarded as unique synapomorphies of the two species. Rather, the similarity between *A. murreeanum* and *A. haemorrhoidale* is better interpreted as an overall resemblance in repeat-unit length and motif organization. This pattern suggests that structural characteristics of the AT-rich region may provide supplementary evidence for phylogenetic proximity within *Acanthosoma*, although broader taxon sampling is still required to test this relationship more rigorously.

The analyses of nucleotide diversity and substitution rates further revealed marked heterogeneity among mitochondrial genes in *A. murreeanum*. In the present study, *ATP8* showed the highest nucleotide diversity as well as the highest Ka and Ka/Ks values, whereas *COX1* was the most conserved gene. This pattern is highly consistent with previous pentatomoid and pentatomid studies, which have repeatedly shown that *ATP8* evolves relatively rapidly, while cytochrome oxidase genes, especially *COX1*, are among the most conserved mitochondrial loci [7,8,10,28]. The Ka/Ks ratios of all 13 PCGs were lower than 1, indicating that these genes are evolving under purifying selection. Together with the absence of gene rearrangement, these results suggest that the mitochondrial genome of *A. murreeanum* has experienced strong functional constraints in its coding regions and overall genomic architecture, whereas the control region represents a more variable component of the mitogenome.

The phylogenetic analyses based on the concatenated mitochondrial dataset recovered congruent topologies under both ML and BI methods and strongly supported a sister relationship between *A. murreeanum* and *A. haemorrhoidale*, with *A. labiduroides* clustering next. This topology supports the monophyly of the sampled species of *Acanthosoma* and provides additional molecular evidence for the placement of *A. murreeanum* within Acanthosomatidae. Previous superfamily-scale and family-level mitogenomic studies have emphasized that increased taxon sampling is essential for stabilizing pentatomoid phylogenetic inference [7,11]. In this context, the inclusion of the mitogenome of *A. murreeanum* expands the molecular data currently available for Acanthosomatidae and improves the basis for congeneric comparison within *Acanthosoma*. The close relationship between *A. murreeanum* and *A. haemorrhoidale* is also broadly consistent with their similar control-region repeat organization, especially the presence of 60 bp tandem-repeat units. Nevertheless, because mitogenomic sampling for Acanthosomatidae remains limited compared with Pentatomidae, the relationship between AT-rich region structure and phylogenetic pattern should be interpreted cautiously. Future studies incorporating additional acanthosomatid mitogenomes and broader Hemiptera sampling will be necessary to determine whether the observed control-region patterns represent genus-level, family-level, or lineage-specific evolutionary signals.

## 5. Conclusions

Overall, the main contribution of this study is not merely the addition of a new mitochondrial genome sequence, but the extension of comparative mitogenomic analysis to a sparsely sampled pentatomoid family. To the best of our knowledge, this study represents the first detailed comparative mitogenomic analysis within the genus *Acanthosoma*, integrating complete mitochondrial genome characterization, gene-wise evolutionary analyses, control-region comparison, and phylogenetic inference. Existing studies have either treated *Acanthosoma* taxa within broader pentatomoid datasets or reported genome resources without such an integrated comparative framework. By combining analyses of genome organization, nucleotide composition, codon usage, tRNA secondary structures, control-region features, gene-specific evolutionary rates, and phylogenetic relationships, the present study shows that several evolutionary patterns repeatedly documented in Pentatomidae—such as strong AT bias, the atypical structure of *trnS1*, the high conservation of *COX1*, and the elevated variability of *ATP8*—are also present in *Acanthosoma*. The absence of gene rearrangement further suggests that *A. murreeanum* retains a conserved mitochondrial genomic architecture, whereas variation in the AT-rich control region provides additional evidence of lineage-specific mitogenomic differentiation. The newly characterized mitogenome of *A. murreeanum* therefore provides not only a new species-level resource, but also a useful comparative framework for future studies of mitochondrial genome evolution and phylogenetic relationships in Acanthosomatidae.

## Figures and Tables

**Table 1:** Nucleotide composition of the mitogenome of *A. murreeanum*.

	A%	T%	G%	C%	A + T%	AT-Skew	GC-Skew
Whole genome	42.56	31.48	11.12	14.84	74.04	0.15	−0.14
PCGs	32.71	40.18	13.26	13.84	72.89	−0.10	−0.02
tRNAs	40.08	37.38	12.69	9.85	77.46	0.03	0.13
rRNAs	32.15	45.73	13.77	8.34	77.88	−0.17	0.25
CR	38.22	35.33	9.87	16.58	73.55	0.04	−0.25

**Table 2:** Mitogenomic organization of *A. murreeanum*.

Genes	Direction	Location		Size (bp)	Intergenic Nucleotides	Codon	
		From	To			Start	Stop
*trnI*	J	1	66	66	0		
*trnQ*	N	64	132	69	−3		
*trnM*	J	141	207	67	8		
*ND2*	J	209	1177	969	1	ATG	TAA
*trnW*	J	1176	1243	68	−2		
*trnC*	N	1236	1300	65	−8		
*trnY*	N	1307	1371	65	6		
*COX1*	J	1376	2911	1536	4	TTG	TAA
*trnL2*	J	2913	2981	69	1		
*COX2*	J	2982	3660	679	0	ATA	T--
*trnK*	J	3661	3732	72	0		
*trnD*	J	3738	3805	68	5		
*ATP8*	J	3806	3964	159	0	ATA	TAA
*ATP6*	J	3958	4626	669	−7	ATG	TAA
*COX3*	J	4628	5416	789	1	ATG	TAA
*trnG*	J	5423	5485	63	6		
*ND3*	J	5486	5836	351	0	ATA	TAA
*trnA*	J	5840	5903	64	3		
*trnR*	J	5904	5971	68	0		
*trnN*	J	5979	6047	69	7		
*trnS1*	J	6047	6115	69	−1		
*trnE*	J	6117	6180	64	1		
*trnF*	N	6179	6247	69	−2		
*ND5*	N	6248	7964	1717	0	ATT	T--
*trnH*	N	7966	8030	65	1		
*ND4*	N	8032	9357	1326	1	ATG	TAG
*ND4L*	N	9351	9638	288	−7	ATT	TAA
*trnT*	J	9641	9705	65	2		
*trnP*	N	9706	9773	68	0		
*ND6*	J	9775	10,269	495	1	ATT	TAA
*CYTB*	J	10,262	11,398	1137	−8	ATG	TAG
*trnS2*	J	11,397	11,465	69	−2		
*ND1*	N	11,489	12,412	924	23	ATT	TAA
*trnL1*	N	12,413	12,482	70	0		
*rrnL*	N	12,483	13,762	1280	0		
*trnV*	N	13,763	13,832	70	0		
*rrnS*	N	13,833	14,614	782	0		
CR		14,615	15,718	1104	0		

Note: ‘J’ means majority strand, and ‘N’ means minority strand. ‘T--’ indicates incomplete stop codon.

**Table 3:** Comparative structural characteristics of the mitochondrial control regions in sampled Acanthosomatidae species.

Species	Mitogenome Length (bp)	Control Region Length (bp)	AT Content (%)	Dominant Tandem Repeat	Other Repeat/Motif Types
*A. haemorrhoidale*	18,923	4328	79.95	60 bp × 20	ATAGA; C(n)GG; poly-A; poly-T; poly-C; (TA)n)
*A. murreeanum*	15,718	1104	73.55	60 bp × 6	ATAGA; C(n)GG; poly-A; poly-T; poly-C; (TA)n)
*A. labiduroides*	16,678	2087	79.92	42 bp × 31	C(n)GG; poly-A; poly-T; poly-C; (TA)n
*Anaxandra taurina*	16,694	2129	79.19	91 bp × 14	ATAGA; C(n)GG; poly-A; poly-T; poly-C; (TA)n
*Sastragala esakii*	15,618	1027	70.89	43 bp × 6	ATAGA; C(n)GG; poly-A; poly-T; poly-C; (TA)n
*Cyphostethus tristriatus*	18,018	3430	78.40	406 bp × 3	ATAGA; C(n)GG; poly-A; poly-T; poly-C; (TA)n
*Microdeuterus* sp.	15,436	845	71.47	52 bp × 2	poly-C; poly-A; poly-T; (TA)n

## Data Availability

The genome sequence data supporting this study are openly available in GenBank of NCBI at https://www.ncbi.nlm.nih.gov (accessed on 4 May 2026) under the accession number PZ323358. The associated BioProject, SRA, and Biosample numbers are PRJNA1369796, SRR38131339, and SAMN57302286, respectively.

## Supplementary Materials

The following supporting information can be downloaded at https://www.mdpi.com/article/10.3390/genes17050560/s1. Figure S1: Relative synonymous codon usage (RSCU) of PCGs in the mitogenomes of Acanthosomatidae; Table S1: Species information used for phylogenetics in this study.
