# Supplementary material for: Complete Mitochondrial Genome of Acanthosoma murreeanum (Hemiptera: Acanthosomatidae): Comparative Analysis and Phylogenetic Implications
Source: Genes (Basel). 2026 May 9;17(5):560. doi: 10.3390/genes17050560 (PMC13205209; doi:10.3390/genes17050560)
Supplement: Supplementary file 1 [file genes-17-00560-s001.zip › Table S1.pdf]

Table S1. Species information used for phylogenetics in this study.

GenBank Number	Organisms	Family	Length	AT%
PZ323358.1	*Acanthosoma murreeanum*	Acanthosomatidae	15718	74.0
OZ334691.1	*Cyphostethus tristriatus*	Acanthosomatidae	18018	75.3
OV884016.1	*Acanthosoma haemorrhoidale*	Acanthosomatidae	18923	75.8
NC_058975.1	*Sastragala esakii*	Acanthosomatidae	15618	75
NC_042801.1	*Anaxandra taurina*	Acanthosomatidae	16694	76.2
MW847242.1	*Microdeuterus* sp.	Acanthosomatidae	15436	74.8
JQ743670.1	*Acanthosoma labiduroides*	Acanthosomatidae	16678	75.7
NC_062724.1	*Brachyrhynchus triangulus*	Aradidae	15170	70.8
NC_030361.1	*Aneurus sublobatus*	Aradidae	16091	67.7
NC_042800.1	*Scoparipes salvazai*	Cydnidae	15173	67.2
NC_058970.1	*Macroscytus subaeneus*	Cydnidae	15853	72.2
NC_072152.1	*Macroscytus japonensis*	Cydnidae	14632	72.7
NC_012457.1	*Macroscytus gibbulus*	Cydnidae	14620	73.8
MF535442.1	*Geotomus* sp.	Cydnidae	15571	73.8
MH643815.1	*Cydnidae* sp.	Cydnidae	15289	73.9
NC_058963.1	*Aethus nigritus*	Cydnidae	15774	72.8
NC_042429.1	*Adrisa magna*	Cydnidae	15935	70.9
NC_042810.1	*Megymenum gracilicorne*	Dinidoridae	15604	72.8
PV423522.1	*Megymenum brevicorne*	Dinidoridae	16889	73.6
PV423523.1	*Eumenotes pacao*	Dinidoridae	16881	74.5
NC_037739.1	*Cyclopelta parva*	Dinidoridae	15422	74.8
NC_069837.1	*Cyclopelta obscura*	Dinidoridae	15426	74.6
NC_086851.1	*Coridius chinensis*	Dinidoridae	16214	75.5
MW899158.1	*Coridius brunneus*	Dinidoridae	15792	75.1
MW619639.1	*Megaris* sp.	Megarididae	15321	76.3

GenBank Number	Organisms	Family	Length	AT%
NC_020373.1	*Dolycoris baccarum*	Pentatomidae	16549	73.3
NC_037724.1	*Dinorhynchus dybowskyi*	Pentatomidae	15952	75.1
NC_037374.1	*Dalsira scabrata*	Pentatomidae	15614	77.6
NC_071749.1	*Chalcopis glandulosa*	Pentatomidae	16534	78.9
NC_085229.1	*Cazira verrucosa*	Pentatomidae	15892	76.4
NC_042805.1	*Caystrus obscurus*	Pentatomidae	15896	77.2
NC_042804.1	*Catacanthus incarnatus*	Pentatomidae	15939	76.6
NC_037741.1	*Carbula sinica*	Pentatomidae	15233	76.4
NC_042802.1	*Brachymna tenuis*	Pentatomidae	15744	77.4
NC_051562.1	*Arma custos*	Pentatomidae	15629	75.8
NC_061538.1	*Anaxilaus musgravei*	Pentatomidae	15951	77
NC_067750.1	*Aelia fieberi*	Pentatomidae	15471	73.6
NC_015342.1	*Megacopta cribraria*	Plataspidae	15647	70.5
NC_067888.1	*Megacopta centronubila*	Plataspidae	15045	73.6
NC_067887.1	*Megacopta caliginosa*	Plataspidae	15641	74.8
NC_067886.1	*Megacopta bituminata*	Plataspidae	15489	76.5
NC_012449.1	*Coptosoma bifarium*	Plataspidae	16179	71.3
NC_058965.1	*Calacta lugubris*	Plataspidae	15731	71
NC_058964.1	*Brachyplatys subaeneus*	Plataspidae	15688	75.2
JQ743675.1	*Poecilocoris nepalensis*	Scutelleridae	14677	77.1
NC_058974.1	*Poecilocoris druraei*	Scutelleridae	16207	73.8
NC_042808.1	*Eurygaster testudinaria*	Scutelleridae	16401	70.8
NC_051942.1	*Chrysocoris stollii*	Scutelleridae	15766	75.5
NC_042803.1	*Cantao ocellatus*	Scutelleridae	15952	74.2
NC_037742.1	*Tessaratoma papillosa*	Tessaratomidae	15565	75.1
NC_053743.1	*Mattiphus splendidus*	Tessaratomidae	15973	72.7

GenBank Number	Organisms	Family	Length	AT%
NC_022449.1	*Eusthenes cupreus*	Tessaratomidae	16229	74.1
NC_058966.1	*Dalcantha dilatata*	Tessaratomidae	15555	76.4
NC_037747.1	*Urostylis flavoannulata*	Urostylididae	15638	74.8
NC_058977.1	*Urolabida histrionica*	Urostylididae	15194	72.9
NC_072983.1	*Urochelellus acutihumeralis*	Urostylididae	16180	74.5
NC_020144.1	*Urochela quadrinotata*	Urostylididae	16587	75.4
NC_072982.1	*Tessaromerus quadriarticulatus*	Urostylididae	15638	76.4
NC_072981.1	*Cobbenicoris guangxiensis*	Urostylididae	15277	76.6
NC_072980.1	*Bannacoris arboreus*	Urostylididae	16603	74.4
