# Supplementary figures and images for: Complete Mitochondrial Genome of Acanthosoma murreeanum (Hemiptera: Acanthosomatidae): Comparative Analysis and Phylogenetic Implications
Source: Genes (Basel). 2026 May 9;17(5):560. doi: 10.3390/genes17050560 (PMC13205209; doi:10.3390/genes17050560)

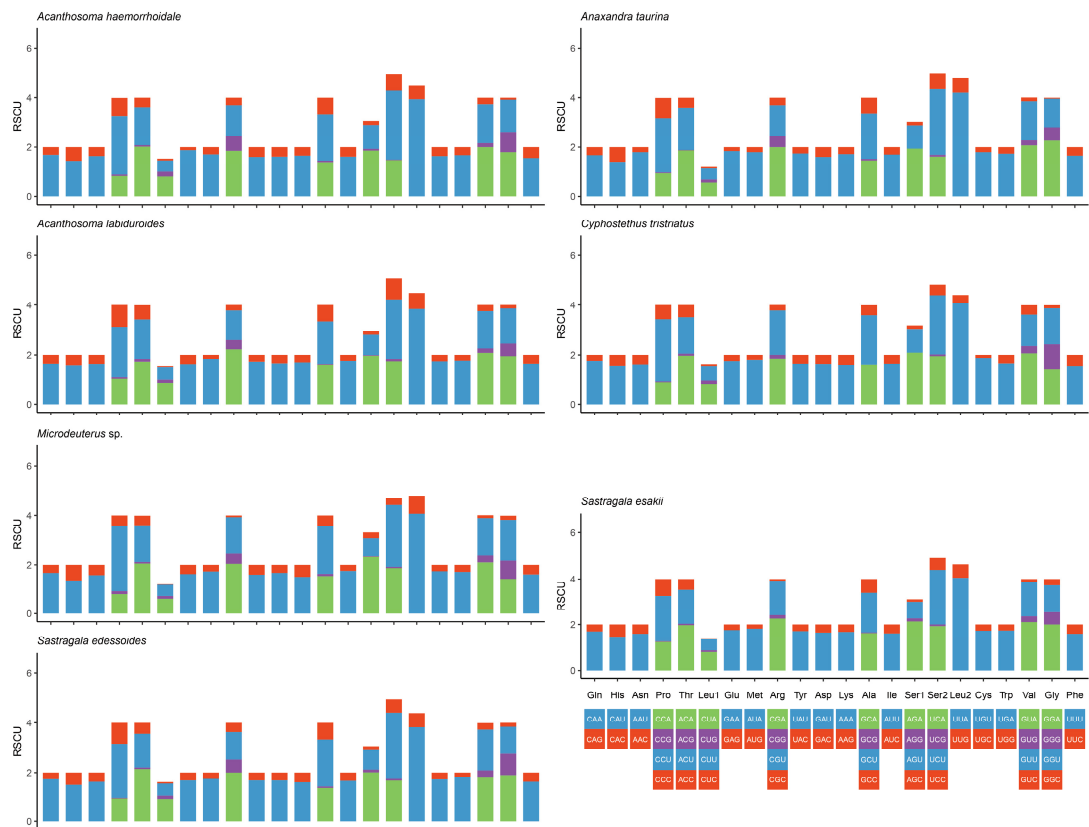

**Figure S1.** Relative synonymous codon usage (RSCU) of PCGs in the mitogenomes of Acanthosomatidae.

Supplement: Supplementary file 1 [file genes-17-00560-s001.zip › Figure S1.pdf]
